# In silico design of multipoint mutants for enhanced performance of *Thermomyces lanuginosus* lipase for efficient biodiesel production

**DOI:** 10.1186/s13068-024-02478-5

**Published:** 2024-02-24

**Authors:** Jinsha Huang, Xiaoman Xie, Wanlin Zheng, Li Xu, Jinyong Yan, Ying Wu, Min Yang, Yunjun Yan

**Affiliations:** Key Laboratory of Molecular Biophysics, Ministry of Education, College of Life Science and Technology, Huazhong University of Science and Technology, Wuhan, People’s Republic of China

**Keywords:** *Thermomyces lanuginosus* lipase, FuncLib, Rosetta Cartesian_ddg, Stability, Catalytic activity, Biodiesel production

## Abstract

**Background:**

Biodiesel, an emerging sustainable and renewable clean energy, has garnered considerable attention as an alternative to fossil fuels. Although lipases are promising catalysts for biodiesel production, their efficiency in industrial-scale application still requires improvement.

**Results:**

In this study, a novel strategy for multi-site mutagenesis in the binding pocket was developed via FuncLib (for mutant enzyme design) and Rosetta Cartesian_ddg (for free energy calculation) to improve the reaction rate and yield of lipase-catalyzed biodiesel production. *Thermomyces lanuginosus* lipase (TLL) with high activity and thermostability was obtained using the *Pichia pastoris* expression system. The specific activities of the mutants M11 and M21 (each with 5 and 4 mutations) were 1.50- and 3.10-fold higher, respectively, than those of the wild-type (wt–TLL). Their corresponding melting temperature profiles increased by 10.53 and 6.01 °C, $T_{50}^{15}$ (the temperature at which the activity is reduced to 50% after 15 min incubation) increased from 60.88 to 68.46 °C and 66.30 °C, and the optimum temperatures shifted from 45 to 50 °C. After incubation in 60% methanol for 1 h, the mutants M11 and M21 retained more than 60% activity, and 45% higher activity than that of wt–TLL. Molecular dynamics simulations indicated that the increase in thermostability could be explained by reduced atomic fluctuation, and the improved catalytic properties were attributed to a reduced binding free energy and newly formed hydrophobic interaction. Yields of biodiesel production catalyzed by mutants M11 and M21 for 48 h at an elevated temperature (50 °C) were 94.03% and 98.56%, respectively, markedly higher than that of the wt–TLL (88.56%) at its optimal temperature (45 °C) by transesterification of soybean oil.

**Conclusions:**

An integrating strategy was first adopted to realize the co-evolution of catalytic efficiency and thermostability of lipase. Two promising mutants M11 and M21 with excellent properties exhibited great potential for practical applications for in biodiesel production.

**Supplementary Information:**

The online version contains supplementary material available at 10.1186/s13068-024-02478-5.

## Highlights

- Biodiesel is a sustainable, renewable and clean energy alternative to fossil fuels.
- Multi-point mutants were created by first integrating FuncLib and Cartesian_ddg.
- M21 achieved 93.37% FAME yield in 24 h, superior to 88.56% of wt–TLL in 48 h.

**Supplementary Information:**

The online version contains supplementary material available at 10.1186/s13068-024-02478-5.

## Background

Owing to the substantial increase in global energy demand and pressing concerns regarding global environmental issues, there is an urgent need for sustainable energy resources [1, 2]. Among the various new energy sources currently available, biodiesel is non-toxic, biodegradable, renewable, green, and clean [3]. Owing to its simple production process and characteristics similar to those of traditional fuels, biodiesel has garnered widespread attention as a high-profile alternative to fossil fuels [4].

Fatty acid methyl esters (FAMEs) are a common type of biodiesel, usually synthesized through transesterification from triacylglycerols (vegetable oils or animal fats) with methanol via chemical [alkaline (e.g., NaOH) and acidic (e.g., HCl and H_2_SO_4_) compounds) or biological (enzymes such as lipase) catalysts [5, 6]. Despite high reaction rates and biodiesel yields, conventional alkaline and acidic catalysts have limitations, such as excessive alcohol requirement, high reaction temperatures, difficulty in separating glycerol, environmental pollution, and equipment corrosion [7]. In contrast, lipase-catalyzed reactions are performed under relatively mild conditions and involve simpler reaction steps, substantially reduced effluent volumes, improved product separation, and higher quality glycerol. Therefore, lipase serves as an eco-friendly substitute for catalyzing biodiesel synthesis [8].

However, the lipase-catalytic reaction system might not consistently demonstrate optimal performance in practical applications [9]. The harsh reaction environment and heat generated by friction in the industrial production process accelerate enzyme inactivation and shorten its operational life span. Additionally, low enzyme activity results in prolonged reaction time and relatively low yield, which ultimately affect the economic viability of biodiesel production [10, 11].

Immobilization and protein engineering are two major strategies to address such bottlenecks. Immobilization technologies have contributed to improvements in performance parameters, including biodiesel yields and recyclability [12, 13]. However, enzymes can be fragile under high-speed stirring conditions. The addition or modification of carriers and procedures for enzyme extraction or purification also pose challenges in terms of environmental protection and production costs [9]. For efficient industrial biodiesel production, protein engineering aimed at improving the activity profiles or heat-tolerance of lipase is a promising strategy [14]. For example, based on a semi-rational evolutionary approach with N-glycosylation in β-sheets of *Rhizomucor miehei* lipase, a mutant N267 was obtained, exhibiting considerably enhanced methanol tolerance and activity over those of the wild-type; in biodiesel synthesis from waste soybean oil at 35 °C, a FAMEs yield of 81.70% was obtained after 48 h [15]. To enhance the stability of lipase from *Geobacillus stearothermophilus* T6, bulky aromatic residues were integrated into the solvent tunnels via rational tunnel engineering. The best mutant A187F/L360F showed an increase in melting temperature (*T*_m_) of 7 °C and achieved 88.00% yield in the biotransformation of waste chicken oil into biodiesel at 45 °C after 24 h [16]. Therefore, modifying one or a few amino acids is an effective strategy to alter the structure and properties of enzymes at minimal cost [17].

The traditional introduction of single-point mutations is not suitable for obtaining significant changes in enzyme properties owing to unpredictable, non-additive effects [18, 19]. A one-shot FuncLib design method has been proposed [20], in which multiple mutations are introduced in the catalytic region to automatically obtain functionally diverse enzyme repertoires, without iterative modeling and experimental validation. Therefore, based on comprehensively sampling sequence space, FuncLib can be utilized to generate lipases that possess suitable properties for industrial production.

Considering that the insertion of multiple mutations in the catalytic pocket may affect protein stability and expression [18, 21], stability assessment is necessary to obtain beneficial variants without compromising structural stability [22, 23]. Rosetta Cartesian_ddg, with relatively high Pearson correlation coefficient of 0.743 among current studies, is widely used to predict potentially stabilizing variants by calculating the overall folding energy change of the protein after mutation [24, 25]. To improve the thermostability of *Streptomyces mobaraenesis* transglutaminase, whole-protein proline virtual scan based on Rosetta Cartesian_ddg was adopted [26]. The *T*_m_ values of single mutation A287P and A265P were, respectively, increased by 2.13 and 0.69 °C. In our previous study [27], Rosetta Cartesian_ddg was utilized to evaluate the impact of mutation on the stability of *Rhizopus oryzae* lipase. The virtual saturated mutant library was reduced from 41 to 17, considerably improve the screening efficiency. The obtained mutant E265V/S267W showed 2.1–4.6 times higher hydrolytic activity than that of wild-type enzyme, while the half-life at 45 °C of E265V/S267W maintains unaltered. It can be seen that Rosetta Cartesian_ddg is an effective tool for protein stability assessment and modification.

*Thermomyces lanuginosus* has a high upper-growth temperature and synthesizes lipase (TLL) with a relatively high optimal temperature and heat-tolerance, compared to those of other microbial lipases. TLL is one of the most commonly used biocatalysts for biodiesel production [28]. Traditional single-point mutations and subsequent combination of the single-point mutations have been used to rapidly obtain TLL mutants with improved performance to conform to the stringent conditions for industrial production [29, 30]. However, there are relatively few studies focusing on multipoint mutagenesis of lipases.

Herein, to rapidly produce enzymes with optimal properties for industrial production, a new strategy incorporating FuncLib and Cartesian_ddg was proposed. First, the FuncLib design method was used to automatically redesign the catalytic region. Subsequently, Rosetta Cartesian_ddg was applied to evaluate the stability of the mutants after functional modification. Finally, mutants with improved catalytic activity and stability were screened and characterized via experimental validation and molecular dynamics (MD) simulations. Applications in biodiesel production were further investigated.

## Results and discussion

### Generation and preliminary screening of FuncLib designs

Structure-guided mutagenesis strategies, such as the combinatorial active-site saturation test (CAST), iterative saturation mutagenesis (ISM), CAST/ISM, and focused rational iterative site-specific mutagenesis (FRISM), have been used for directed evolution experiments to obtain diverse catalytic activities, requiring high-throughput analytical methods to screen mutant libraries [31–34]. As an alternative, FuncLib is an efficient algorithm for the prediction of stable and functionally diverse mutants with less experimental effort by directly introducing multiple point mutations into the enzyme pocket in a single shot [20]. Herein, two FuncLib runs were conducted to predict variants harboring 2–5 mutations.

**Fig. 1:**
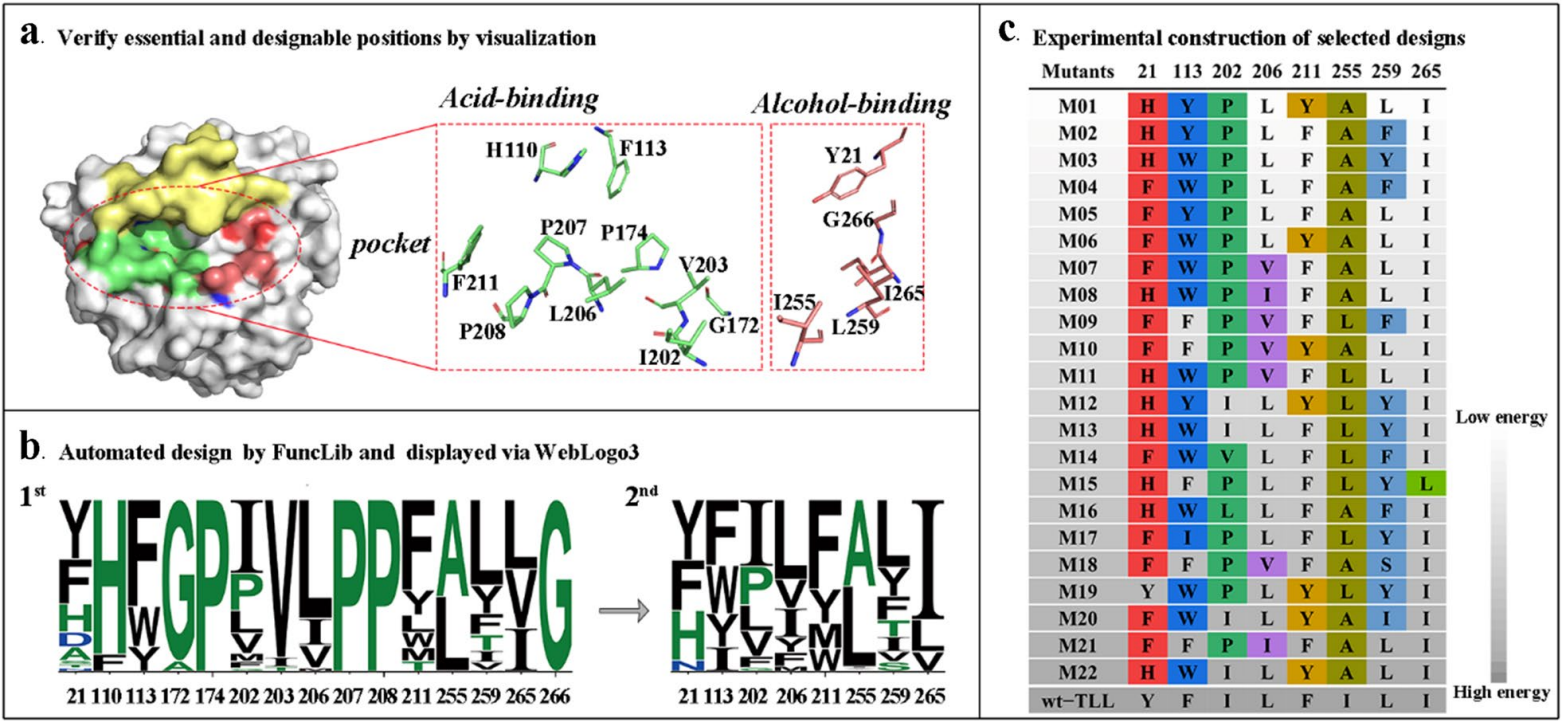
Flowchart of the key steps in the computational redesign of the active site of TLL. **a** Architecture of the substrate binding sites in TLL. The yellow surface represents the residues located in the lid of the open conformation. The active site residues located in the nucleophilic (alcohol-binding) and electrophilic (acid-binding) pockets are represented in chartreuse and salmon, respectively. **b** Multipoint mutants in two rounds of redesign are exhaustively enumerated using an automated web-accessible server, FuncLib (https://funclib.weizmann.ac.il/bin/steps), and represented using WebLogo (https://weblogo.threeplusone.com/) to generate a sequence logo. The height of each letter represents the substitution probability of the amino-acid residue [35]. **c** The designs were ranked by energy, and designs with low energy were selected for experimental construction. Mutations in the same designed position are shown in consistent colors

On the basis of an enzyme–ligand (OLA) complex (PDB entry 1gt6), residues within 5 Å from the substrate were considered to make up the catalytic region and identified using PyMol (-select access_channel, byres OLA around 5). To ensure normal catalytic function, amino acids in the significant functional domains, such as catalytic triad (S146, D201, H258), oxyanion hole (G82, S83), lid region (residues 83–95), and consensus sequence GXSXG (residues 144–148) were maintained unaltered in the calculation. Consequently, fifteen residues that constitute the main part of the channel were initially targeted as the starting point for randomization in the first FuncLib run (FuncLib1, 1st). As shown in the FuncLib1 subfile in Additional file 1, 1000 candidates were generated and all exhibited lower total_score than that of wt–TLL (serial number 010101010101010101010101010101 in the subfile). Then, the occurrences of mutations in targeted positions were analyzed by WebLogo 3. As shown in Fig. 1a, ten amino acids (H110, F113, G172, P174, I202, V203, L206, P207, P208, and F211) were located in the acid-binding area, and the other five (Y21, I255, L259, I265, and G266) were located in the alcohol-binding area. The computational result for the first run is shown in Fig. 1b (left, 1st), the prevalent amino acids at positions 174, 207, 208, and 266 were constant (Pro, Pro, Pro, and Gly, respectively), and His, Gly, and Val residues were heavily preferred at positions 110, 172, and 203. To reduce the number of mutant sites and shrink the mutant library, these seven sites were neglected and the remaining eight positions (21, 113, 202, 206, 211, 255, 259, and 265) were used in the second FuncLib mutagenesis (FuncLib2, 2nd). Likewise, 1000 candidates of lower total_score than that of wt–TLL (serial number 0101010101010101) were generated (see FuncLib2 subfile in Additional file 1), and were subjected to Rosetta Cartesian_ddg to minimize adverse effects of the insertion of multiple mutations in the catalytic pocket on protein stability and expression [18, 21]. The substitution probability of the amino-acid residue in FuncLib2 is shown in Fig. 1b (right, 2nd), and hydrophobic residues were preferred at these sites. Additionally, by analyzing the data of folding free energy change in the Cartesian_ddg subfile in Additional file 1, 321 out of 1000 candidates showed non-negative values. Therefore, according to the rank in FuncLib2, twenty-two top-ranked mutants from the remaining 679 FuncLib2 designs were manually selected for analyses of catalytic characteristics. The mutant sites are illustrated in Fig. 1c.

### Qualitative screening of active mutants

As mutations in the substrate binding channel influence enzymatic activity [23, 36], active substitutions were first qualitatively screened via a halo-formation assay using rhodamine B-olive oil dishes. As shown in Additional file 2: Fig. S1, mutations had negative effects on 11 variants (M04, M05, M06, M08, M09, M13, M14, M17, M19, M20, and M20), as evidenced by the lack of a halo surrounding the corresponding colonies.

A red halo appeared around the colonies of other 11 candidates (M01, M02, M03, M07, M10, M11, M12, M15, M16, M18, and M21), indicating that these variants displayed lipolytic activity. Owing to it was difficult to maintain the same number of cells for each mutant, the size of red halos was slightly varied in the same mutant [37, 38]. Importantly, the size of halo generated is closely related to the enzymatic activity [39], the colonies with larger red halos in mutants M01, M02, M03, M07, M10, M11, M12, M15, M16, M18, and M21 were hand-picked for subsequent experimental verification.

### Analysis of the enzymatic activity of mutants

Supernatants of equal volume from individual flasks were separated by 15% SDS-PAGE, revealing the expected molecular weight (35 kDa) (Additional file 2: Fig. S2). The single band indicated that the fermentation supernatant can be used directly for subsequent characterization without additional purification.

**Fig. 2:**
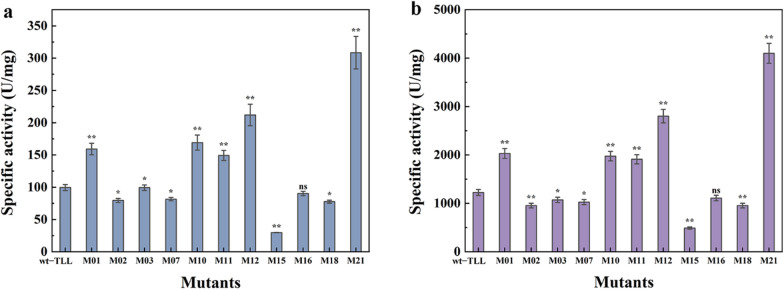
Determination of enzymatic activity using **a** *p*NPP and **b** olive oil as substrates at 45 °C and pH 8.0 in Tris–HCl buffer. The error bars in graphs represent standard deviations (*n* = 3). The symbols indicate significant differences between mutants and wild-type: ns (no significant difference), *p* > 0.05; *, *p* < 0.05; **, *p* < 0.01

To further determine the enzymatic activity of the mutants, the divergence in hydrolytic activity among the positive FuncLib designs was determined on 4-nitrophenyl palmitate (*p*NPP) and olive oil (Fig. 2). As shown in Fig. 2a, the mutants M01, M10, M11, M12, and M21 displayed improved hydrolytic activity with *p*NPP as a substrate (i.e., 1.60- 1.70-, 1.50-, 2.13-, and 3.10-fold higher than that of the wild-type, respectively). The activities of other six mutants (M02, M03, M07, M15, M16, and M18) were severely reduced. Similar results were obtained for catalytic activity assay on olive oil (Fig. 2b).

### Kinetic properties of wt–TLL and mutants

**Fig. 3:**
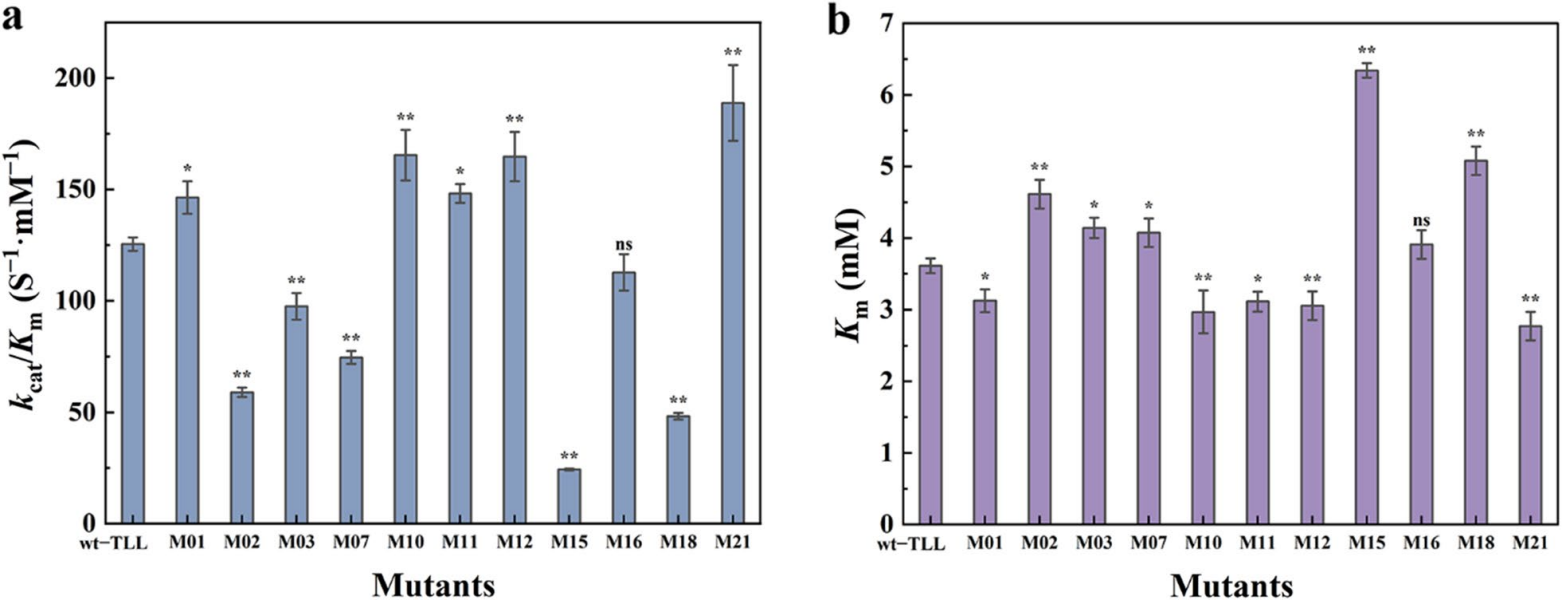
Kinetic parameters of wild-type and mutant TLL determined using a series of *p*NPP concentrations (10 mM to 100 mM) at 10 mM intervals as a substrate. **a** Catalytic efficiency (*k*_cat_/*K*_m_), and **b** *K*_m_ values. The error bars in graphs represent standard deviations (*n* = 3). The symbols indicate significant differences between mutants and wild-type: ns (no significant difference), *p* > 0.05; *, *p* < 0.05; **, *p* < 0.01

Kinetic parameters (*K*_m_, *V*_max_, and *k*_cat_/*K*_m_) of wt–TLL and the mutants were further investigated. As shown in Fig. 3a, the *k*_cat_/*K*_m_ value for wt–TLL was 125.40 ± 3.01 s^−1^ mM^−1^, whereas the corresponding values for the mutants M01, M10, M11, M12, and M21 were increased to 146.29 ± 7.30, 165.33 ± 11.40, 148.11 ± 4.20, 164.67 ± 11.10, and 188.80 ± 17.00 s^−1^ mM^−1^, respectively, implying an improved catalytic efficiency. These results were consistent with those for enzymatic activity assay (Fig. 2). *K*_m_ values are negatively correlated with substrate affinity [40]. As illustrated in Fig. 3b, the *K*_m_ values of the mutants M01, M10, M11, M12, and M21 were 3.13 ± 0.16, 2.97 ± 0.30, 3.11 ± 0.14, 3.05 ± 0.20, and 2.77 ± 0.20 mM, respectively, remarkably lower than that of wt–TLL (3.61 ± 0.10 mM), suggesting a higher substrate affinity. For the other six mutants (M02, M03, M07, M15, M16, and M18), the opposite trend was observed, with increased *K*_m_ values and decreased *k*_cat_/*K*_m_ values, indicating a diminished affinity and reaction rate.

### Analysis of binding free energy based on the MM/PBSA method

To elucidate the complex effects of mutations on the enzyme–substrate (*p*NPP) interaction, the binding free energies and separate contributions were calculated according to the MM/PBSA method [41]; the values are reported in Table 1. Negative values represent a favorable binding process [42], and lower values indicate stronger bonds and a higher probability that a reaction will occur [43]. For M01, M10, M11, M12, and M21, the binding energies were 11.86, 9.62, 8.95, 29.02, and 15.32 kJ/mol lower than that of wt–TLL (− 97.72 kJ/mol), showing enhanced binding affinity to the substrate. The other six mutants (M02, M03, M07, M15, M16, and M18) displayed reduced affinity with increased values (32.42, 0.86, 10.60 10.39, 4.65, and 3.65 kJ/mol, each higher than that of wt–TLL). These results were consistent with the *K*_m_ results. The mutants M01, M10, M11, M12, and M21 with enhanced binding affinity were selected for subsequent thermostability analyses.

**Table 1:** Binding free energy and individual energy components for wild-type and mutant TLL with *p*NPP based on molecular mechanics/Poisson–Boltzmann surface area calculations

Wild-type/mutants	Binding energy (kJ/mol)	van der Waals energy (kJ/mol)	Electrostatic energy (kJ/mol)	Polar solvation energy (kJ/mol)	Non-polar solvation energy (kJ/mol)
Wt–TLL	− 97.72	− 129.45	− 29.74	80.914	− 19.449
M01	− 109.58	− 145.73	− 27.09	84.10	− 20.85
M02	− 65.28	− 89.02	− 13.29	52.89	− 15.85
M03	− 96.86	− 115.96	− 17.06	54.25	− 18.08
M07	− 87.12	− 121.86	− 26.64	80.12	− 18.74
M10	− 107.34	− 142.42	− 20.22	75.90	− 20.61
M11	− 106.67	− 136.62	− 24.45	73.88	− 19.48
M12	− 126.74	− 158.28	− 4.15	57.79	− 22.10
M15	− 87.33	− 119.22	− 28.00	77.93	− 18.04
M16	− 93.07	− 122.50	− 11.36	60.89	− 20.11
M18	− 94.07	− 116.94	− 7.03	47.42	− 17.53
M21	− 113.04	− 148.56	− 23.65	80.46	− 21.30

### Thermal stability of the mutants M01, M10, M11, M12, and M21

**Fig. 4:**
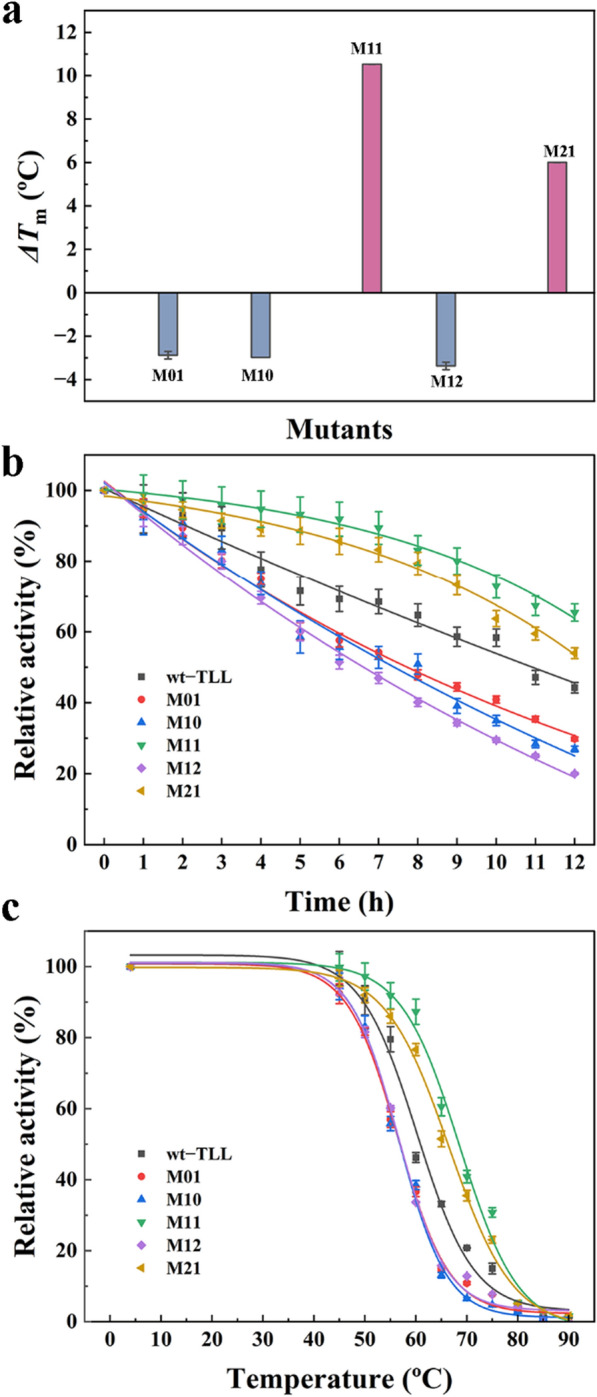
Thermostability during enzymatic hydrolysis. **a** Melting temperature changes (*ΔT*_m_) of wild-type and mutant TLL measured using differential scanning fluorimetry. **b** Thermodynamic curve of wild-type and mutant TLL incubated at 50 °C for different times. **c** The thermal deactivation of wild-type and mutant TLL at different temperatures for 15 min heating treatment ($T_{50}^{15}$). The error bars in graphs represent standard deviations (*n* = 3)

To verify the effect of accumulated mutations on structural stability, *T*_m_ values were investigated using the DSF method (Fig. 4a). The melting temperature changes (*ΔT*_m_) of M01, M10, and M12 were − 2.88, − 2.98, and − 3.37 °C, respectively, indicating a poor contribution to stability. The mutants M11 and M21 displayed favorable stability with *T*_m_ values 10.53 and 6.01 °C higher than that of wt–TLL.

The thermal inactivation process was evaluated by testing residual activity after specific exposure times at 50 (Fig. 4b). Mutants with positive *ΔT*_m_ values (M11 and M21) exhibited improved heat resistance, whereas the others (M01, M10, and M12) displayed worse stability than the wt–TLL. After 12 h of incubation at 50 °C, the relative activity of the wt–TLL was 44%, whereas M11 and M21 retained 65% and 54% of the enzyme activity and M01, M10, and M12 displayed only 30%, 27%, and 20%, respectively.

$T_{50}^{15}$ is another important parameter for assessing the kinetic stability of enzyme molecules. As seen in Fig. 4c, the $T_{50}^{15}$ of wt–TLL was 60.88 °C. The $T_{50}^{15}$ of mutants M01, M10 and M12 with negative *ΔT*_m_ were reduced to 56.86, 56.88 and 56.94 °C, showing poor heat resistance. Whereas, the better mutants M11 and M21 exhibited 7.58 and 5.42 °C improvement in $T_{50}^{15}$, indicating a significant improvement in kinetic stability over the wild-type protein.

### Biochemical properties of beneficial variants

**Fig. 5:**
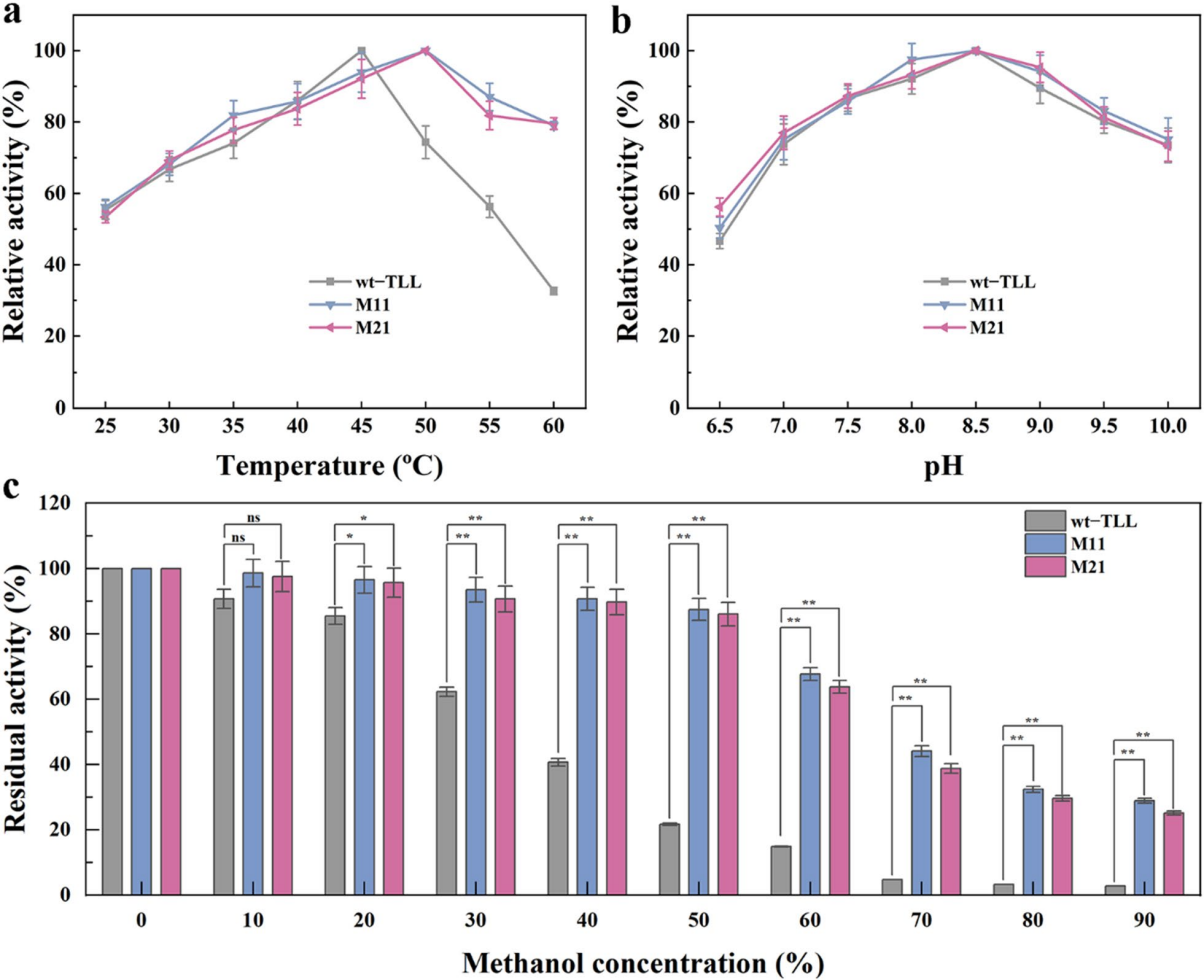
Comparison of enzymatic properties between wt–TLL and positive mutants (M11 and M21). **a** Optimum temperature for wt–TLL and the mutants M11 and M21. **b** Optimal pH for wt–TLL and the mutants M11 and M21. **c** The methanol tolerance of wt–TLL and the mutants M11 and M21 determined by analyzing the residual activity of enzymes preincubated in methanol at various concentrations (10% to 90%) for 1 h at their respective optimum temperatures. The error bars in graphs represent standard deviations (*n* = 3). The symbols indicate significant differences between mutants and wild-type: ns (no significant difference), *p* > 0.05; *, *p* < 0.05; **, *p* < 0.01

To identify the effect of multiple mutations in the catalytic sites on the optimal temperature, the activities of wt–TLL, M11, and M21 were measured at various temperatures (25–60 °C). As shown in Fig. 5a, the optimal temperatures of the mutants M11 and M21 increased from 45 to 50 °C. At higher temperature (55–60 °C), the relative activities of the mutants were no less than 80%, whereas the relative activity of the wt–TLL did not exceed 70%. Hence, the introduction of stable mutations in TLL pockets resulted in sustained higher activity at elevated temperature.

TLL is a eukaryotic lipase with high alkaline tolerance, and its optimum pH ranges from 8.0 to 10.0 [30]. As depicted in Fig. 5b, although M11 and M21 exhibited higher activity under weakly alkaline conditions than wt–TLL, there was no significant difference in their optimal pH (8.5). These results indicated that mutations in the active site have little effect on the optimal pH of TLL.

In addition, TLL is widely used to prepare biodiesel with natural oil and short-chain alcohols (generally methanol) [12]. Thus, it is essential to investigate its methanol tolerance. As depicted in Fig. 5c, the denaturation effect of methanol on the enzyme was observed in a concentration-dependent manner. M11 and M21 consistently displayed higher activity under the same pretreatment. Moreover, after pre-incubation in 60% methanol solution for 1 h, the residual activities of M11 and M21 were all > 60%, whereas wt–TLL nearly lost its activity (retained only 15% activity). Overall, both M11 and M21 showed good resistance to methanol, enabling them suitable for biodiesel production.

### Biodiesel production

**Fig. 6:**
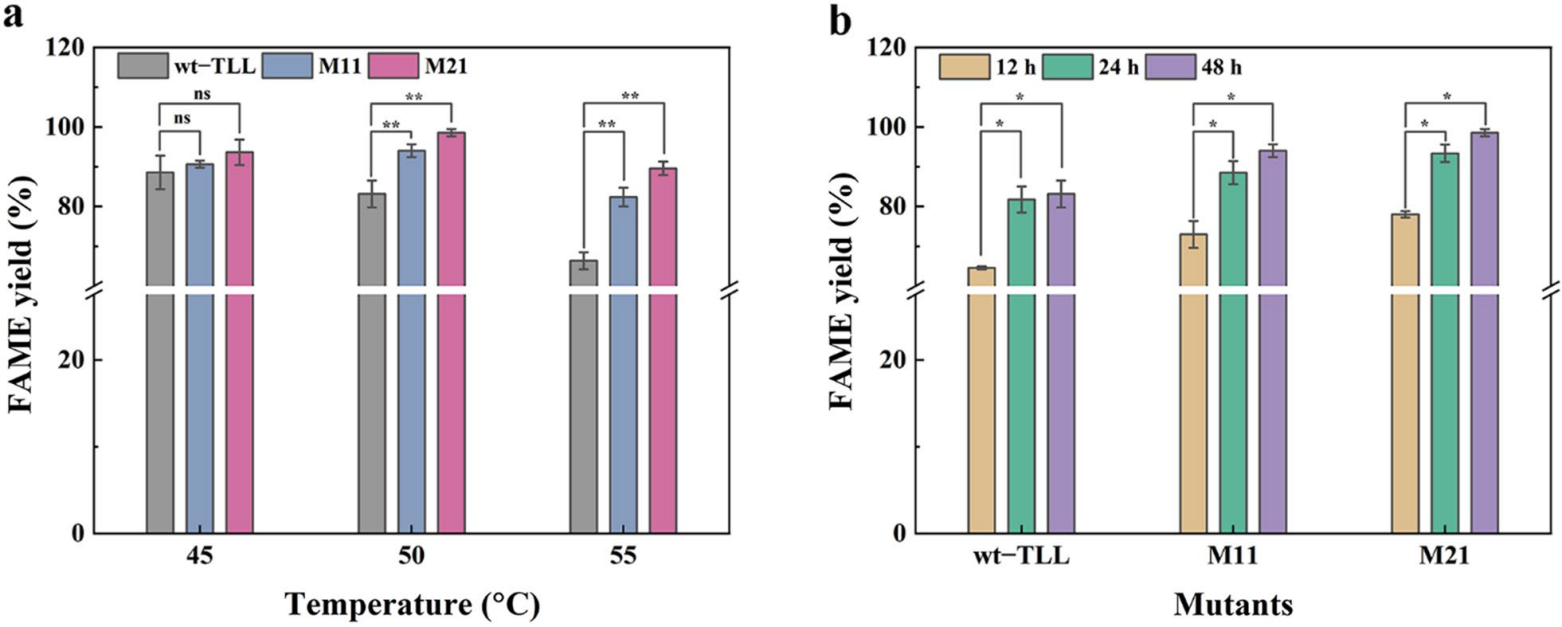
Comparison of the FAME yield between wt–TLL and positive mutants (M11 and M21). **a** Transesterification of soybean oil at 45, 50, and 55 °C after 48 h. **b** Transesterification of soybean oil at 50 °C for different times (12, 24, and 48 h). The error bars in graphs represent standard deviations (*n* = 3). The symbols indicate significant differences between mutants and wild-type: ns (no significant difference), *p* > 0.05; *, *p* < 0.05; **, *p* < 0.01

Lipases with high catalytic efficiency and heat- and methanol-tolerance are effective catalysts for biodiesel synthesis to avoid denaturation or deactivation by alcohols and to obtain a higher conversion rate [15]. With satisfactory catalytic performances, M11 and M21 could yield higher conversion rates in one-step synthesis of biodiesel from soybean oil. As shown in Fig. 6a, the yields of wt–TLL were 88.56%, 83.15%, and 66.38% after 48 h at temperatures of 45, 50, and 55 °C, respectively. Under the same conditions, M21 converted 93.64%, 98.56%, and 89.59% of soybean oil into biodiesel, showing the best performance. FAME yields of 90.64%, 94.03%, and 82.36% were obtained for M11, wherein the values were slightly lower than that of mutant M21. The mutants M11 and M21 displayed higher conversion rates than wt–TLL at all tested temperature, and this could likely be attributed to the enhanced catalytic activity and thermostability of the mutants [30]. Meanwhile, as increased temperature leads to the loss of stability and durability [44], the FAME yields could not be further increased, even by prolonging the reaction time. Therefore, the yields of wt–TLL and both mutants dropped at higher temperature (55 °C). As M11 and M21 had a more robust structure rigidity and higher thermostability than wt–TLL, the decrease in FAME yield was more pronounced in wt–TLL. Furthermore, transesterification of soybean oil with wt–TLL, M11 and M21 at 50 °C at intervals was also tested (Fig. 6b). The FAME yields of M11 and M21 were 73.01% and 78.03%, respectively, within 12 h, which were 1.13- and 1.21-fold higher than that of wt–TLL (64.61%). After 24 h, wt–TLL achieved 81.73% FAME yield, whereas M11 and M21 exhibited excellent performance, achieving approximately 90% FAME yield (88.50% and 93.37%), indicating that improving the catalytic activity and stability of TLL had a positive effect on biodiesel production.

To further assess the ability of the mutants in biodiesel production, two waste oils with different acid values were taken as different type of feedstock. As seen in Additional file 2: Fig. S3, the wild-type and mutant TLL could convert big part of waste greases into biodiesel after 48 h. Mutants M11 and M21 exhibited better performance than wt–TLL owing to their higher thermostability and catalytic activity. The FAME yields of M11 were, respectively, 70.30% and 76.80%. During the same period, M21 converted 80.50% and 86.49% of waste greases into biodiesel, showing the best performance. Whereas, wt–TLL performed the worst, only obtaining 61.37% and 65.59% FAME yields at 48 h. With reference to soybean oil (Fig. 6), the reaction time was much longer and the yields were lower when using waste oils as feedstock. The phenomenon may be attributed to two possible reasons. In one hand, the high amount of free fatty acids in waste oil has a specific toxic effect on the enzyme, leading to a decrease in enzyme activity [45]. On the other hand, transesterification and esterification reactions occur simultaneously, reducing the transesterification efficiency of lipases [15, 29]. Consequently, it would take a relatively long time to reach equilibrium in biodiesel production with waste greases as substrate.

According to recent studies, more than 90% conversion can be obtained for biodiesel production using lipases as biocatalysts at temperatures between 30 and 50 °C [6, 46]. However, the reaction time and conversion rate are affected by the intrinsic properties of the lipases [7, 47, 48]. To date, substantial efforts have been made to facilitate their applications in biodiesel production. Immobilization is an effective technology for maximizing efficiency and economic feasibility, and more than 90% conversion can be obtained for biodiesel production within 24 h [49–51]. However, the costly carriers, complicated preparation of the immobilized enzymes, and isolation of their products have limited the industrial-scale application [52].

Enzyme engineering strategies have been widely tried (Table 2). By iterative saturation mutagenesis (ISM) against high B-factor residues, the double mutant TLL-S105C/D27R of TLL achieved 81.00% FAME yield in the biotransformation of waste grease, which was higher than that of wild-type TLL (67.00%) [29]. Furthermore, surface charge engineering was also adopted on TLL to enhance the industrial application. The obtained mutant D165E exhibited the highest biodiesel conversion yield of 93.56% [53]. Additionally, MD simulations and Discovery Studio have been used to improve the thermostability and biodiesel yields of TLL. The mutant G91C showed a 5 °C increase in the optimum temperature and achieved 91.00% yield in the biotransformation of *Cornus wilsoniana* oil into biodiesel at 43 °C after 60 h [30]. Although a great number of protein engineering methods have been explored to enhance biodiesel production to some extent, the yields at nearly 100% are still necessary to make the enzymatic process competitive with chemical processes [46].

**Table 2:** Overview of previous studies on use of lipases in biodiesel production

Organism	Expression system	Lipase	Biodiesel feedstock	Yield (%)	Ratio (oil:methanol)	Temperature (°C)	Time (h)	References
*Thermomyces lanuginosus*	*Pichia pastoris* KM71H	G91C	*Cornus wilsoniana* oil	91.00	1:5	43	60	[30]
*Thermomyces lanuginosus*	*Escherichia coli*	S105C/D27R	Waste grease	90.00	1:3	30	24	[29]
*Rhizomucor miehei*	*Pichia pastoris* GS115	N314	Waste soybean oil	70.50	1:6	35	24	[55]
*Rhizomucor miehei*	*Pichia pastoris* GS115	N267	Waste soybean oil	81.70	1:6	35	24	[15]
*Rhizomucor miehei*	*Pichia pastoris* GS115	N218	Colza oil	90.46	1:6	35	24	[56]
*Geobacillus stearothermophilus*	*Escherichia coli* BL21	A187F/L360F	Waste chicken oil	88.00	1:5	45	24	[16]
*Geobacillus stearothermophilus*	*Escherichia coli* BL21	H86Y/A269T/R374W	Waste chicken oil	64.00	1:4.5	45	24	[57]
*Yarrowia lipolytica*	*Escherichia coli* BL21	YLIP9L1Bp3	Palm oil	~ 40.00	1:5	50	24	[58]
*Yarrowia lipolytica*	*Yarrowia lipolytica*	lipase 2	Soybean oil	91.80	1:4	40	24	[59]
*Thermomyces lanuginosus*	*Pichia pastoris* GS115	M21	Soybean oil	98.56 (93.37)	1:4	50	48 (24)	This study

Herein, using a dual rational design strategy based on FuncLib and Cartesian_ddg, mutations in the binding pocket of TLL were found to have positive effects on thermostability and activity, resulting in a higher conversion efficiency and shorter reaction duration. The biodiesel yields of M11 and M21 reached 94.03% and 98.56%, respectively, from 5 g of oil after 48 h. In particular, M21 exhibited the best performance, achieving 93.37% FAME yield within 24 h. A one-step methanol feeding protocol has also been used here, which can simplify the production process [15, 54]. Furthermore, as the targeted lipase was secreted extracellularly in *P. pastoris*, the fermentation supernatant could be used directly as a biocatalyst without purification, which can greatly reduce the production cost and be beneficial for biodiesel production.

In addition, a process integrating deep eutectic solvent and ultrasonic agitation in scale-up reactor was developed and successfully applied for the liquid lipase (*Yarrowia lipolytica* Lipase 2) catalyzed synthesis of biodiesel in our lab [48]. Following this method, the FAME yield can achieve over 99% with a reaction time of 6 h at 40 °C. Therefore, in combination with the aforementioned liquid lipase-catalytic system in the subsequent study, it can be predicted that the designed TLL mutants with high enzymatic activity and heat- and methanol-tolerance can obtain even higher conversion rate in a shorter reaction duration and exhibit better potential for industrial-scale application of biodiesel.

Besides deep investigation on lipases performing effectively in biodiesel green production, there are other processes needing further researches to realize the sustainability of biofuels according to life cycle analysis, such as utilization of renewable resources (biomass), good agricultural practices at the cultivation stage, and efficient logistics and transportation [60, 61].

### Mechanisms underlying the enhanced properties of TLL mutants

**Fig. 7:**
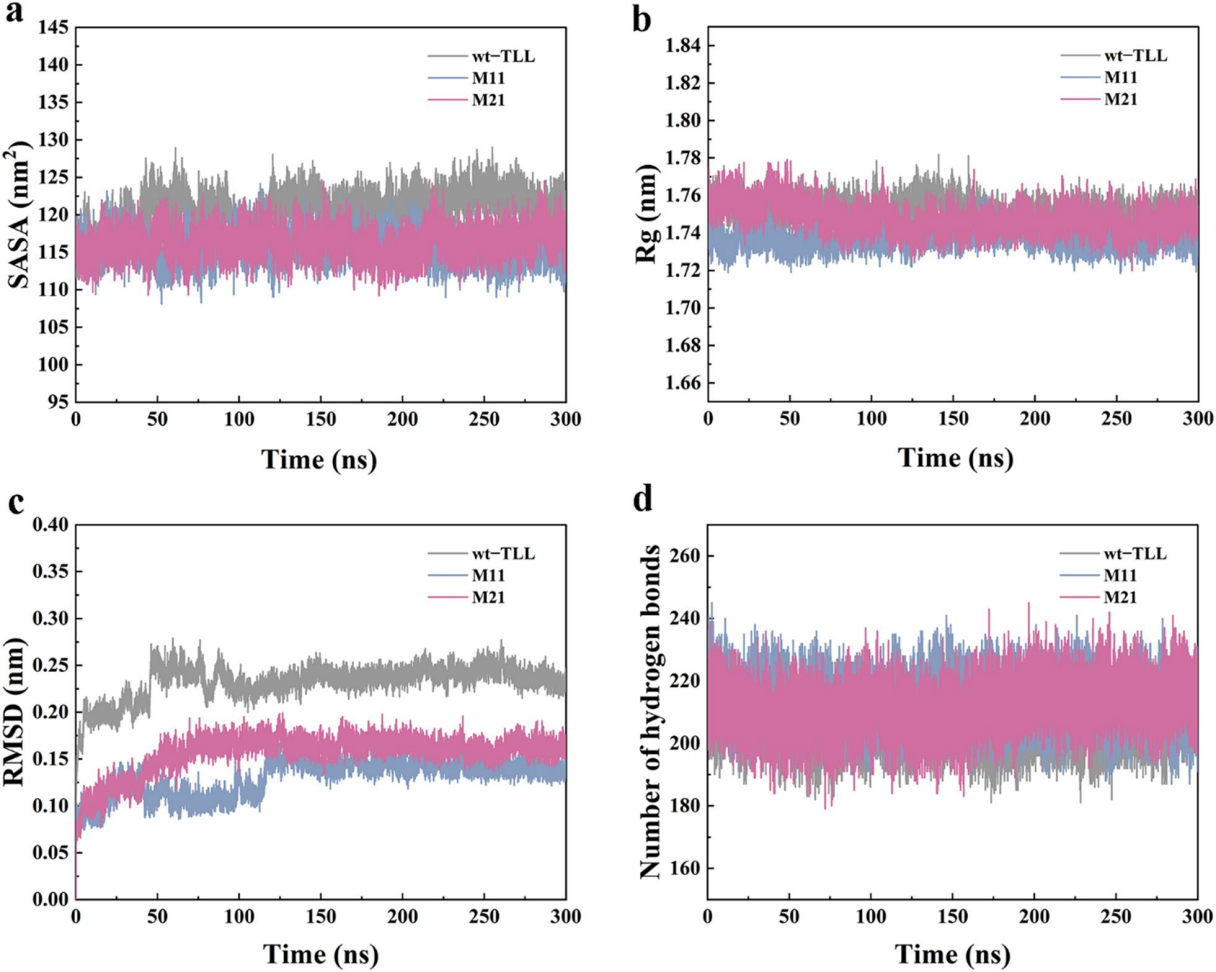
Structural analysis of wt–TLL and the mutants M11 and M21 determined by 300-ns MD simulations (100 ns) at 353 K. **a** Solvent-accessible surface area (SASA). **b** Average radius of gyration (Rg). **c** Root-mean-square deviation (RMSD) of protein Cα atoms. **d** Changes in the number of hydrogen bonds

The structures of M11 and M21 were generated using FuncLib, revealing a perfectly conserved typical α/β-hydrolase fold. To elucidate the effects of multiple mutations on thermostability, the structural characteristics (RMSD, SASA, Rg, number of hydrogen bonds, and secondary structure) were investigated using MD simulations at 353 K for 300 ns (Fig. 7).

SASA correlates with protein aggregation, and a smaller SASA value indicates a tighter structure [44]. At 353 K, the average SASA values of wt–TLL was 120.13 ± 0.83 nm^2^, while the average SASA values of M11 and M21 were reduced to 116.02 ± 0.57 and 116.76 ± 0.68 nm^2^, respectively (Fig. 7a, Additional file 2: Table S2). Similarly, Rg is negatively associated with structural robustness [44]. As shown in Fig. 7b and Additional file 2: Table S2, the Rg value of M11 and M21 stayed approximately around 1.737 ± 0.000 and 1.743 ± 0.008 nm, slightly lower than that of the wt–TLL (1.749 ± 0.005 nm). The decreased SASA and Rg values indicated that the introduction of mutations in the binding pocket made the structures of M11 and M21 more compact and robust. The types and contents of secondary structures of M11 and M21 were also analyzed as illustrated in Additional file 2: Table S3. The unaltered types of secondary structures demonstrated that all mutants folded correctly as wt–TLL. Furthermore, the percentage distribution of strand for M11 and M21 were respectively 33.61% and 33.99%, higher than that of wt–TLL (33.14%). The percentages distribution of helix for M11 and M21 were respectively increased from 29.29 to 30.68% and 29.86%. The changed in the contents of secondary structures of M11 and M21 may also contribute to the improved stability.

RMSD is a key parameter to estimate thermal fluctuations in proteins by predicting the conformational changes of the protein backbone during simulation, which is negatively related to structural rigidity. As shown in Fig. 7c, the RMSD of wt–TLL and the mutants displayed similar trends and were stable in the last 150 ns. These results indicated that the binding of the substrate at the catalytic site was stable and that mutations did not change the backbone stability. The RMSD value of wt–TLL was approximately 0.239 nm during the 300 ns simulations, whereas the mutants M11 and M21 displayed more stable structures owing to the 0.096 and 0.081 nm reduction (Additional file 2: Table S2). Accordingly, the RMSD analysis indicated that multiple stabilizing mutations were introduced into M11 and M21 without affecting stability. Furthermore, the number of hydrogen bonds was calculated using gmx_h bond (Fig. 7d and Additional file 2: Table S2). The average number of bonds was higher for M11 (214) and M21 (213) than for wt–TLL (208), suggesting that the newly formed hydrogen bonds were responsible for the enhanced thermostability.

The interaction between the active pocket and substrate determines enzyme activity [62]. To elucidate the mechanism underpinning the enhanced catalytic activity and increased yield of biodiesel production for M11 and M21, the molecular docking experiments towards the model substrate (*p*NPP) and the main fatty acids (palmitic, oleic and linoleic acids) consisting in soybean oil were performed. The docking binding energies for the selected docking conformations were illustrated in Additional file 2: Table S4. Taking *p*NPP as the ligand, molecular docking experiments were first performed between wild-type, the mutants of TLL and *p*NPP. The docking binding energies of wt–TLL, M11 and M21 were, respectively, − 4.27, − 4.54, and − 4.62 kJ/mol, indicating all were stable docking conformations. Additionally, the binding energies towards the main fatty acids of soybean oil were all negative (Additional file 2: Table S4), suggesting wild-type and the mutants of TLL also had good binding affinity to palmitic, oleic and linoleic acids. In particular, the binding energies of M11 and M21 were much lower than that of wt–TLL, demonstrating that the accumulation of hydrophobic mutations could provide a beneficial hydrophobic environment and stronger binding affinity.

**Fig. 8:**
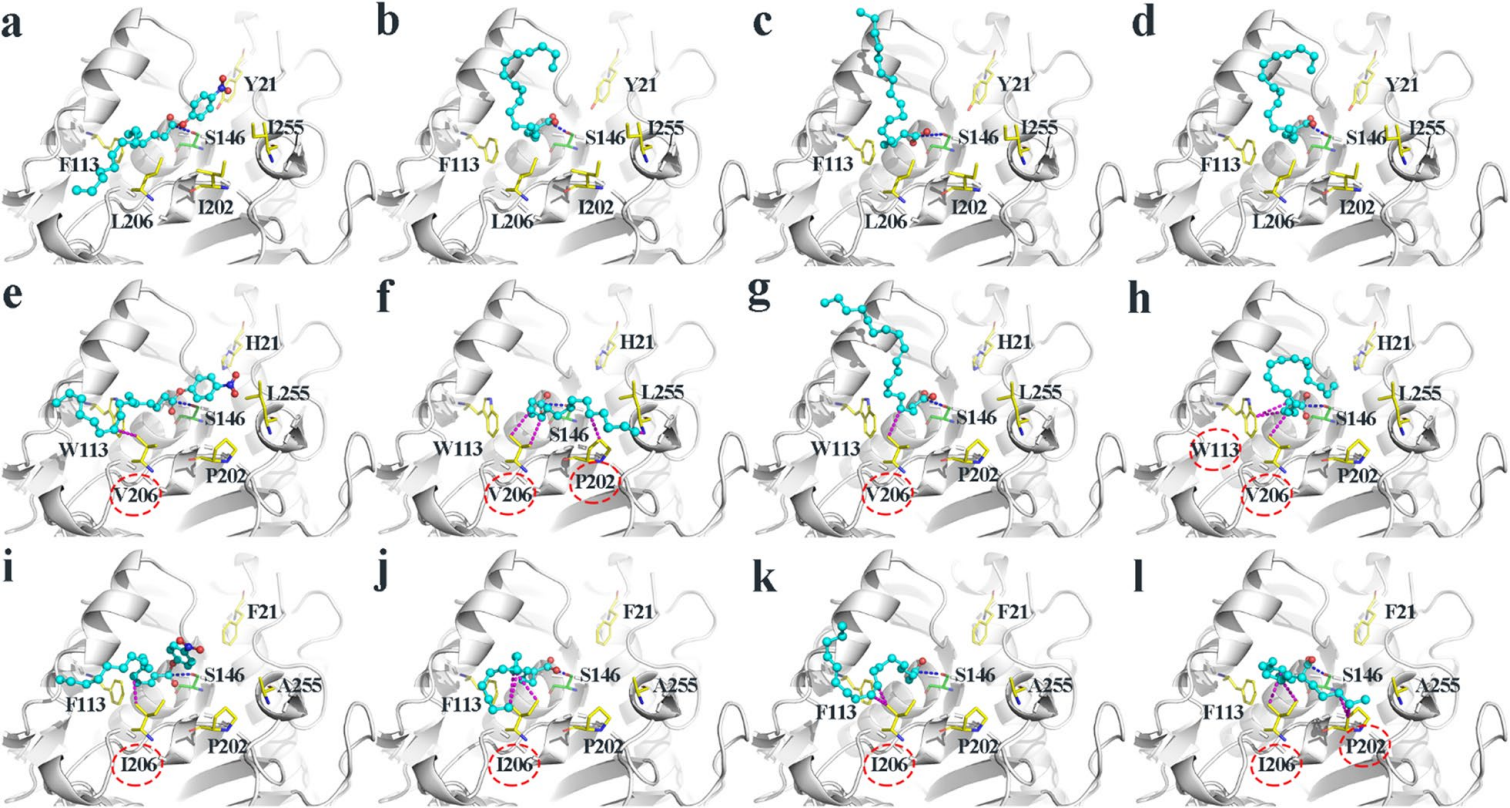
Structural analysis of the interaction between the main contents of soybean oil (palmitic, oleic and linoleic acids) and the mutant amino-acid residues in the binding site of wt–TLL, the mutant M11, and the mutant M21. **a** wt–TLL, *p*NPP; **b** wt–TLL, palmitic acid; **c** wt–TLL, oleic acid; **d** wt–TLL, linoleic acid; **e** M11, *p*NPP; **f** M11, palmitic acid; **g** M11, oleic acid; **h** M11, linoleic acid; **i** M21, *p*NPP; **j** M21, palmitic acid; **k** M21, oleic acid; **l** M21, linoleic acid. The catalytic S146 are colored in green and shown as sticks. The target residues for mutagenesis are colored in yellow and shown as sticks. The substrates are colored in cyan and shown as ball and sticks. The blue dashed line indicates the hydrophilic attack distance. The magenta dashed line indicates the interaction

Due to five and four mutations being observed in mutants M11 and M21 at sites 21, 113, 202, 206, and 255, respectively (Fig. 1c), the structural analysis of the interaction between various ligands and the above-mentioned mutant amino-acid residues was visualized in Fig. 8. It was well known that the occurrence of mutations in the catalytic region may affect the binding process, leading to changes in the structure, flexibility, and conformational dynamics of the enzyme and substrate [44]. Thus, the conformation of the enzyme was redistributed during the dynamics of catalysis and the orientation of the substrate varies from different enzyme–ligand complexes [63].

In the mutant M11–*p*NPP system (Fig. 8e), a hydrophobic interaction was created between V206 and the carbon chain of *p*NPP, which was also observed in the mutant M21–*p*NPP system with an L–to–I change at residue 206 (Fig. 8i); whereas, this phenomenon was not apparent in the wt–TLL–*p*NPP system (Fig. 8a). A new apolar interaction detected after the mutation further improved the catalytic activity of lipase by influencing substrate binding and product release [27, 64]. Thus, a certain degree of increase in the hydrophobic interaction of the substrate binding channel could improve the catalytic efficiency of lipase toward hydrophobic substrates.

Moreover, the binding conformations with the main fatty acids of soybean oil (palmitic, oleic and linoleic acids) were further analyzed. In the M11 systems (Fig. 8f–h), with a W-to-F change at residue 113, one hydrophobic interaction was created only towards the carbon chain of linoleic acid (Fig. 8h) and with an I-to-P change at residue 202, one hydrophobic interaction was created only towards the carbon chain of palmitic acid (Fig. 8f); while with an L-to-V change at residue 206, two hydrophobic interactions were created towards the carbon chain of palmitic acid (Fig. 8f), and only one hydrophobic interaction was created towards the carbon chain of oleic acid (Fig. 8g) and linoleic acid (Fig. 8h). Likewise, in the M21 systems (Fig. 8j–l), extra hydrophobic interactions were similarly observed. With an I-to-P change at residue 202, two hydrophobic interactions were created only towards the carbon chain of linoleic acid (Fig. 8l), and with an L-to-I change at residue 206, 3, 2 and 2 hydrophobic interactions were respectively created towards the carbon chain of palmitic acid (Fig. 8j), oleic acid (Fig. 8k) and linoleic acid (Fig. 8l). Similarly, such phenomena were not apparent in the wt–TLL system (Fig. 8b–d). Therefore, in comparison with wt–TLL, the better performance in biodiesel production of mutants M11 and M21 were probably attributed to their extra hydrophobic interactions with their substrates, soybean oils.

## Conclusions

Collectively, a dual-strategy using FuncLib and Cartesian_ddg was applied to alter the binding pocket of TLL to improve its enzymatic properties, making it promising for biodiesel production by increasing reaction rates and yields. The activity and heat-tolerance of the positive mutants M11 and M21 were significantly enhanced because of the addition of hydrophobic bonds, which reduced atomic fluctuations and improved affinity. The specific activities of the positive mutants M11 and M21 were increased by 50% and 210% (Fig. 2a), their *T*_m_ values were increased by 10.53 and 6.01 °C (Fig. 4a), and their $T_{50}^{15}$ values were increased by 7.58 and 5.42 °C (Fig. 4c) in comparison with those of wt–TLL, respectively. The mutants shortened the reaction time, simplified the production process, and successfully improved the efficiency of biodiesel production. The FAME yield of mutant M21 was 93.37% and 98.56% at a higher temperature at 24 and 48 h, respectively, which were considerably higher than that of wt–TLL (81.73% and 83.15%). The simple approach proposed in the study facilitates the optimization of enzyme performance and provides a model for development of efficient catalysts for economical and sustainable biodiesel production.

## Methods

### Gene, plasmid, strains, and growth conditions

The gene encoding wild-type TLL (wt–TLL) was provided in our laboratory. The plasmid pGAP815α was constructed on the basis of pAO815 by substituting the AOX promoter with GAP and fusing an evolved signal peptide from *Saccharomyces cerevisiae* [65] (Additional file 2: Fig. S4). The GAP promoter avoids the multiple addition of methanol during incubation and reduces the risk of bacterial contamination. The *Pichia pastoris* expression system was selected owing to the low heteroprotein secretion; the introduction of signal peptides enables the target protein to be secreted extracellularly, avoiding an unnecessary purification process. *Escherichia coli* DH5α and *P. pastoris* GS115 were used as host strains for vector construction and protein expression. *E. coli* was cultured in Luria–Bertani (LB) medium (5 g L^−1^ yeast extract, 10 g L^−1^ tryptone, and 10 g L^−1^ sodium chloride) with 100 μg mL^−1^ ampicillin. *Pichia pastoris* was grown in yeast extract–peptone–dextrose (YPD) medium (20 g L^−1^ glucose, 10 g L^−1^ yeast extract, and 20 g L^−1^ tryptone) and BMMY medium (10 g L^−1^ yeast extract, 20 g L^−1^ tryptone, 100 mM potassium phosphate buffer, 13.4 g L^−1^ yeast nitrogen base, 0.4 mg L^−1^ biotin, and 5 mL L^−1^ methanol).

### Computational enzyme design

The crystal structure of TLL (PDB entry 1dte) was used as an input file for computational enzyme design. FuncLib was used to predict mutants with enhanced activity and Rosetta Cartesian_ddg was used to evaluate the stability of the mutants.

Mutated positions were obtained in the ligand (OLA) access channel of a nearly identical structure (PDB entry 1gt6, with an S-to-A change at residue 146) using PyMol (-select access_channel, byres OLA around 5). Notably, residues in critical domains, such as the catalytic triad (S146, D201, H258), oxyanion hole (G82, S83), lid region (residues 83–95), and consensus sequence GXSXG (residues 144–148), were unaltered in the calculation.

With the TLL crystal structure (PDB entry 1dte) as an input, FuncLib calculations (https://funclib.weizmann.ac.il/) were performed twice (FuncLib1 and FuncLib2) for active-site redesign. The sequence space for each site was explored using PSSM and Rosetta energy filters with default parameters, with filtering based on the following thresholds: PSSM ≥ − 2 and ∆∆G ≤ ± 6 R.e.u [66]. All potential FuncLib designs harboring 2–5 mutations were automatically ranked based on the all-atom Rosetta energy function. WebLogo 3 (https://weblogo.threeplusone.com/) was used to graphically represent the occurrences of mutations in targeted positions by analyzing sequences of the top 1000 ranked designs. The height of each letter represents the substitution probability of the amino-acid residue [35].

Because the insertion of numerous mutations into the catalytic region may reduce protein stability [21, 66], the Rosettta Cartesian_ddg stability prediction method was applied to evaluate the effect of mutations on the stability of FuncLib2 designs [24]. A positive value indicates that the mutations decrease the stability of the enzyme. The code for Rosetta Cartesian_ddg is described in our previous research [27].

Finally, the 22 top-ranked FuncLib2 designs with negative folding-free energy changes were selected from the library for further experimental analyses. The FuncLib and Cartesian_ddg results are available in Additional file 1.

### Construction of FuncLib variants

The gene sequences of FuncLib designs were amplified by numerous overlap extension PCRs and inserted into the *Eco*RI/*Not*I-digested (Takara, Beijing, China) vector pGAP815α via homologous recombination [27]. Thereafter, the *Sal*I-linearized (Takara, Beijing, China) plasmids were integrated into the genome of *P. pastoris* strain GS115 [67]. All primers used in the study are listed in Additional file 2: Table S1.

### Qualitative detection of lipase activity using rhodamine B-olive oil dishes

The BMMY–rhodamine B-olive oil plates were prepared according to previously described methods [68], using BMMY solid medium that contained 8 mg of rhodamine B and 10 mL of emulsified olive oil (Olivoila, Shanghai, China) per liter. Only active colonies can catalyze the hydrolysis reaction of olive oil and display clear red halos after a few days of growth. Moreover, the size of the halo correlates with the activity of recombinants [68]. In the stage, 14 colonies for each recombinant protein were first selected from the minimal dextrose solid Petri dishes and then transferred onto such plates for phenotype verification. Notably, the recombinant strain GS115/pGAP815α/wt–TLL and GS115 were used as positive and negative controls, respectively.

### Heterologous expression of FuncLib variants in *P. pastoris*

Recombinant strains harboring visible halos were picked and incubated in 5 mL of YPD medium for 24 h at 28 °C. Then, the seed cultures were inoculated into a 500-mL triangle flask with 50 mL of BMMY medium and incubated in a shaker for 96 h at 28 °C to produce proteins. The molecular weights of TLL in the supernatant were confirmed by sodium dodecyl sulfate polyacrylamide gel electrophoresis (SDS-PAGE) using 15% precast mini polyacrylamide gels, and the gels were stained using the eStain® LG Protein Staining System (Genscript, Nanjing, China). Protein concentrations were determined using the Bradford Protein Assay Kit (Tiangen Biotech, Beijing, China).

### Determination of lipase activity

(i) **Titration method.** The activity of TLL mutants was first measured using a titration method [69]. The total system was composed of 4 mL of emulsified mixture (1 mL of olive oil and 3 mL of 2% [m/v] polyvinyl alcohol solution), 5 mL of Tris–HCl buffer (50 mM, pH 8.5), and 1 mL of fermentation supernatant. The reaction was performed in a shaking water bath for 10 min and terminated with 15 mL of cold acetone/ethanol (1:1, v/v). Amounts of fatty acids released were verified by titration with 50 mM NaOH in the presence of phenolphthalein as an indicator. As a control, an identical volume of Tris–HCl was used instead of the enzyme. One unit (U) of lipase activity was defined as the consumption of enzyme to liberate 1 μmol fatty acid per minute.
(ii) **Colorimetric method.** The activity of TLL mutants was determined using a colorimetric method [70]. The system contained 10 μL of *p*NPP (50 mM, dissolved in acetonitrile solution), 40 μL of absolute ethyl alcohol, 10 μL of fermentation supernatant, and 940 μL of Tris–HCl buffer (50 mM, pH 8.5). Hydrolysis of the substrate was monitored via spectrophotometry. Each unit (U) of activity was defined as the amount of enzyme required to produce 1 μmol *p*-nitrophenol per min. As a control, an equivalent volume of Tris–HCl buffer was used instead of the enzyme solution.

### Analysis of kinetic parameters

Kinetic parameters (*k*_cat_, *K*_m_, and *k*_cat_/*K*_m_) were investigated by estimating the initial reaction rates against a series of *p*NPP concentrations (10–100 mM) at 10 mM intervals. Reactions were performed at 45 °C with reference to the colorimetric method. *K*_m_ and *V*_max_ values were calculated following the classical Michaelis–Menten model [40], and the catalytic constant (*k*_cat_) was calculated using *V*_max_ values and the molecular mass of TLL (35 kDa).

### Characteristics of thermostability

(i) **Determination of *T***_**m**_**.** Differential scanning fluorimetry (DSF), a thermo-analytical technique, is widely used to estimate the *T*_m_ value during the unfolding process [36]. The *T*_m_ value is defined as the temperature with the maximal fluorescence change rate (dRFU/dT). The enzyme solution was mixed with SYPRO orange dye (5000×, Thermo Fisher Scientific, USA), followed by centrifugation (2000×*g*) for 2 min at 4 °C. *T*_m_ values were obtained by slowly heating the mixture from 25 to 90 °C at a rate of 0.3 °C per min in a StepOnePlus real-time PCR system (Applied Biosystems, Waltham, MA, USA) [27].
(ii) **Thermal inactivation analysis.** 1 mL of the fermentation supernatant of the samples (wild-type or mutant TLL) were incubated in a water bath at 50 °C for 12 h. Then, 10 μL of the samples were withdrawn at specified intervals and then chilled on ice. The residual activity at each time point was determined using the *p*NPP-based colorimetric method [27], and the relative activity at time zero was treated as 100%. The data were fitted to an exponential function by nonlinear regression using Origin 2021 [71].

Additionally, the $T_{50}^{15}$ value is defined as the temperature at which the enzyme activity is reduced to 50% after 15-min incubation at various temperatures (4, 45, 50, 55, 60, 65, 70, 75, 80, 85 and 90 °C). Then, the activities were quantitatively measured with reference to the *p*NPP-based colorimetric method. The specific activity at 45 °C was considered as 100% and $T_{50}^{15}$ values were calculated by fitting the residual activities at certain temperatures to a Boltzmann sigmoidal function by nonlinear regression using Origin 2021 [72, 73].

### Optimization of reaction conditions

To investigate the optimum reaction temperature, the catalytic activities of wild-type and mutant TLL were detected at temperatures ranging from 25 to 60 °C with 5 °C intervals using the colorimetric method. For a given enzyme, the relative activity at the optimum temperature was treated as 100%. At the optimum temperature, the pH activity profiles of wild-type and mutant TLL were tested at pH values from 6.5 to 10.0 with 0.5 intervals using the colorimetric method. For a given enzyme, the relative activity at the optimum pH was considered 100%.

### Evaluation of methanol tolerance

The reaction samples (wild-type or mutant TLL) were first incubated in a methanol solution with various concentrations ranging from 10 to 90% with 10% intervals for 1 h at 25 °C. The residual activity of the enzymes was measured against *p*NPP based on the colorimetric method at their own optimal temperatures (45, 50, and 50 °C). The activity of the untreated sample was set to 100%, and the residual activity at each point was defined as the percentage of the initial activity.

### Transesterification of greases into FAMEs

(i) **Production of biodiesel.** The transesterification reactions of soybean oil and waste greases were preformed according to our previous study [59]. The system was composed of 5 g of soybean oil (COFCO, Beijing, China), 925 µL of methanol (oil-to-methanol molar ratio of 1:4), 5 mL of fermentation supernatant, and 1.3 mL of Tris–HCl buffer (pH 8.5, 50 mM) mixed in a 50 mL conical flask with a cover. Methanol was added in a one-step feeding process, and the reaction was performed in a thermostatic oscillation incubator at 200 rpm. The effect of temperature on the transesterification reaction of soybean oil was measured at 45, 50, and 55 °C for 48 h. And the effect of reaction time at 50 °C on the transesterification reaction of soybean oil was measured at 12, 24, and 48 h; additionally, the production of FAME from two waste oils was also analyzed for 48 h at 50 °C. The acid values of waste oil (1) and waste oil (2) were 296.70 and 290.00 mg KOH/g, respectively.
(ii) **Quantitative analysis of FAMEs.** Gas chromatography was performed using methyl heptadecanoate (Sigma-Aldrich, Shanghai, China) as the internal standard [48]. The GC 9790 Plus instrument (Fuli, Zhejiang, China) equipped with a flame-ionization detector and an Agilent HP-INNOWAX capillary column (30 m × 0.25 mm × 0.25 µm; J & W Scientific, Folsom, CA, USA) were used. The initial temperature of the column was set at 180 °C, increased to 230 °C at a rate of 3 °C/min, and maintained for 3 min. The temperatures of the injector and detector were set at 230 and 280 °C, respectively. The FAMEs content was calculated using the area normalization method [48], and FAMEs yield (%) was defined as the ratio of the total quantity of methyl ester produced to the theoretical maximum quantity of fatty acids in the crude oil before the reaction.

Additionally, buffers of different pH values were prepared in accordance with the temperatures used. All experimental determinations were replicated three times and the results were expressed as means ± standard deviations. Statistical significance was analyzed by one-way ANOVA and Duncan’s test using Graphpad Prism version 8 software program. The level of significance with *p* > 0.05 (ns, no significant difference), *p* < 0.05 (*) and *p* < 0.01 (**) was deemed statistically significant.

### In silico MD simulations

MD simulations were conducted under GROMACS software (version 2021.1) on a Linux-based workstation using *p*NPP as the ligand. The structures of variants utilized were generated by the FuncLib calculation, and the PDB entry of the wt–TLL used was 1dte. The topology of ligand *p*NPP was generated using the ACPYPE webserver (https://www.bio2byte.be/acpype/submit/) [74], and the topology of the protein and protein–ligand complex was built using GROMACS software. MD simulations for wild-type and mutant systems were performed under the same conditions.

(i) MD simulations of protein at 353 K. Each system was performed twice. The initial structure was immersed into a cubic box and solvated in TIP3P water models, and an excess of Na^+^ or Cl^−^ was added to electrostatically neutralize the system. Then, the solvated and electroneutral system was subjected to a steepest descent energy minimization of 5000 steps (2 fs each steep) under the conditions of the Amber99sb-ILDNforce field to remove the structure conflicts. After energy minimization, position restraint simulations were conducted under 100-ps NVT (constant number of particles, volume, and temperature) and 100-ps NPT (constant number of particles, pressure, and temperature) equilibration. Afterwards, the MD production for each system was run for 300 ns with periodic boundary conditions and a time-step of 2 fs. Finally, post-processing and analysis were performed using standard GROMACS tools with coordinates stored in 2-ps time-steps [15]. The root-mean-square deviation (RMSD), average radius of gyration (Rg), solvent-accessible surface area (SASA), content of secondary structures, and number of hydrogen bonds were calculated to explore the effect of mutations on thermostability.
(ii) MD simulations of protein–ligand complex at 328 K. To gain deeper insight into the influence of mutations on enzyme–substate interactions, MD simulations of protein–ligand complex were conducted at 328 K for 100 ns and the binding free energies were estimated using the molecular mechanics/Poisson–Boltzmann surface area (MM/PBSA) approach according to the last 10-ns trajectories [43].

### Semiflexible molecular docking using AutoDock Vina

The composition and content of fatty acids in the used soybean was 12.44% palmitic acid, 1.53% stearic acid, 22.82% oleic acid, 55.89% linoleic acid, and 7.32% linolenic acid. Semiflexible molecular docking experiments were conducted with AutoDock Vina, using the model substrate *p*NPP and the main contents of soybean oil (palmitic, oleic and linoleic acids) as ligands [75]. The interaction networks and the binding mode of the enzyme–substrate complex were constructed and visualized using PyMOL.

### Supplementary Information

**Additional file 1: FuncLib1.** The original data calculated in the first FuncLib run. **FuncLib2.** The original data calculated in the second FuncLib run. *Cartesian_ddg.* Folding free energies of FuncLib2 designs predicted by Cartesian_ddg.

**Additional file 2: Table S1.** Primers used for multipoint mutagenesis introduction in the study. **Table S2.** The average values of SASA, Rg, RMSD, and number of hydrogen bonds. **Table S3.** Average contents of secondary structures in wt–TLL and the mutants M11 and M21 determined via MD simulations (100 ns) at 353 K. **Table S4.** The docking binding energies for selected docking conformations calculated by molecular docking. **Figure S1.** Screening of lipolytic activity of recombinant TLL on rhodamine B-olive oil agar plate under visible light. (1) mutant M01, (2) mutant M02, (3) mutant M03, (4) mutant M04, (5) mutant M05, (6) mutant M06, (7) mutant M07, (8) mutant M08, (9) mutant M09, (10) mutant M10, (11) mutant M11, (12) mutant M12, (13) mutant M13, (14) mutant M14, (15) mutant M15, (16) mutant M16, (17) mutant M17, (18) mutant M18, (119) mutant M19, (20) mutant M20, (21) mutant M21, (22) mutant M22, (23) wt–TLL, (24) parent *Pichia pastoris*, GS115. **Figure S2.** Determination of protein molecular weight of wild and mutant TLL on SDS-PAGE using 15% precast mini polyacrylamide gel. Lane M: standard protein molecular weight (180/140/100/75/60/45/35/25/15/10 kDa), lane 1: parent *P. pastoris* GS115, lane 2: mutant M01, lane 3: mutant M02, lane 4: mutant M03, lane 5: mutant M07, lane 6: mutant M10, lane 7: mutant M11, lane 8: mutant M12, lane 9: mutant M15, lane 10: mutant M16, lane 11: mutant M18, lane 12: mutant M21, lane 13: wt–TLL. **Figure S3.** Biotransformation of waste oils catalyzed to FAME by wt–TLL and positive mutants (M11 and M21) for 48 h. The error bars in graphs represent standard deviations (*n* = 3). The asterisks indicate significant differences between mutants and wild-type: *, *p* < 0.05; **, *p* < 0.01. **Figure S4.** The schematic diagram of recombinant plasmid pGAP815α/TLL including TLL gene with 6×His-tag in N-terminal, GAP promoter and an evolved signal peptide from yeast *Saccharomyces cerevisiae*.

## Data Availability

All data generated or analyzed of this article are included within the article and its additional files.

## Abbreviations

DSF: Differential scanning fluorimetry
FAME: Fatty acid methyl ester
MM/PBSA: Molecular mechanics/Poisson–Boltzmann surface area
MD: Molecular dynamics
NPT: Number of particles, pressure, and temperature
NVT: Number of particles, volume, and temperature
RMSD: Root-mean-square deviation
SASA: Solvent-accessible surface area
TLL: *Thermomyces lanuginosus* lipase
YPD: Yeast extract–peptone–dextrose
LB: Luria–Bertani
$T_{50}^{15}$: Temperature at which enzymes lose 50% activity after heat treatment for 15 min
