# Supplementary material for: In silico design of multipoint mutants for enhanced performance of Thermomyces lanuginosus lipase for efficient biodiesel production
Source: Biotechnol Biofuels Bioprod. 2024 Feb 24;17:33. doi: 10.1186/s13068-024-02478-5 (PMC10894483; doi:10.1186/s13068-024-02478-5)
Supplement: Supplementary file 2 — Additional file 2: Table S1. Primers used for multipoint mutagenesis introduction in the study. Table S2. The average values of SASA, Rg, RMSD, and number of hydrogen bonds. Table S3. Average contents of secondary structures in wt–TLL and the mutants M11 and M21 determined via MD simulations (100 ns) at 353 K. Table S4. The docking binding energies for selected docking conformations calculated by molecular docking. Figure S1. Screening of lipolytic activity of recombinant TLL on rhodamine B-olive oil agar plate under visible light. (1) mutant M01, (2) mutant M02, (3) mutant M03, (4) mutant M04, (5) mutant M05, (6) mutant M06, (7) mutant M07, (8) mutant M08, (9) mutant M09, (10) mutant M10, (11) mutant M11, (12) mutant M12, (13) mutant M13, (14) mutant M14, (15) mutant M15, (16) mutant M16, (17) mutant M17, (18) mutant M18, (119) mutant M19, (20) mutant M20, (21) mutant M21, (22) mutant M22, (23) wt–TLL, (24) parent Pichia pastoris, GS115. Figure S2. Determination of protein molecular weight of wild and mutant TLL on SDS-PAGE using 15% precast mini polyacrylamide gel. Lane M: standard protein molecular weight (180/140/100/75/60/45/35/25/15/10 kDa), lane 1: parent P. pastoris GS115, lane 2: mutant M01, lane 3: mutant M02, lane 4: mutant M03, lane 5: mutant M07, lane 6: mutant M10, lane 7: mutant M11, lane 8: mutant M12, lane 9: mutant M15, lane 10: mutant M16, lane 11: mutant M18, lane 12: mutant M21, lane 13: wt–TLL. Figure S3. Biotransformation of waste oils catalyzed to FAME by wt–TLL and positive mutants (M11 and M21) for 48 h. The error bars in graphs represent standard deviations (n = 3). The asterisks indicate significant differences between mutants and wild-type: *, p < 0.05; **, p < 0.01. Figure S4. The schematic diagram of recombinant plasmid pGAP815α/TLL including TLL gene with 6×His-tag in N-terminal, GAP promoter and an evolved signal peptide from yeast Saccharomyces cerevisiae. [file 13068_2024_2478_MOESM2_ESM.docx]

**Additional file 2**

# *In silico* design of multipoint mutants for enhanced performance of *Thermomyces lanuginosus* lipase for efficient biodiesel production

Jinsha Huang, Xiaoman Xie, Wanlin Zheng, Li Xu^*^, Jinyong Yan, Ying Wu, Min Yang, Yunjun Yan^*^

Key Laboratory of Molecular Biophysics, Ministry of Education, College of Life Science and Technology, Huazhong University of Science and Technology, Wuhan, People’s Republic of China

^*^Corresponding authors:

Li Xu, xuli@hust.edu.cn

Yunjun Yan, yanyunjun@hust.edu.cn

Additional materials captions

**Table S1** Primers used for multipoint mutagenesis introduction in the study

**Table S2** The average values of SASA, Rg, RMSD, and number of hydrogen bonds

**Table S3** Average contents of secondary structures in wt−TLL and the mutants M11 and M21 determined via MD simulations (100 ns) at 353 K

**Table S4** The docking binding energies for selected docking conformations calculated by molecular docking

**Fig. S1** Screening of lipolytic activity of recombinant TLL on rhodamine B−olive oil agar plate under visible light. (1) mutant M01, (2) mutant M02, (3) mutant M03, (4) mutant M04, (5) mutant M05, (6) mutant M06, (7) mutant M07, (8) mutant M08, (9) mutant M09, (10) mutant M10, (11) mutant M11, (12) mutant M12, (13) mutant M13, (14) mutant M14, (15) mutant M15, (16) mutant M16, (17) mutant M17, (18) mutant M18, (119) mutant M19, (20) mutant M20, (21) mutant M21, (22) mutant M22, (23) wt−TLL, (24) parent *Pichia pastoris*, GS115.

**Fig. S2** Determination of protein molecular weight of wild and mutant TLL on SDS‑PAGE using 15% precast mini polyacrylamide gel. Lane M: standard protein molecular weight (180/140/100/75/60/45/35/25/15/10 kDa), lane 1: parent *P. pastoris* GS115, lane 2: mutant M01, lane 3: mutant M02, lane 4: mutant M03, lane 5: mutant M07, lane 6: mutant M10, lane 7: mutant M11, lane 8: mutant M12, lane 9: mutant M15, lane 10: mutant M16, lane 11: mutant M18, lane 12: mutant M21, lane 13: wt−TLL.

**Fig. S3** Biotransformation of waste oils catalyzed to FAME by wt–TLL and positive mutants (M11 and M21) for 48 h. The error bars in graphs represent standard deviations (n = 3). The asterisks indicate significant differences between mutants and wild-type: *, *p* < 0.05; **, *p* < 0.01.

**Fig. S4** The schematic diagram of recombinant plasmid pGAP815α/TLL including TLL gene with 6×His‑tag in N‑terminal, GAP promoter and an evolved signal peptide from yeast *Saccharomyces cerevisiae*.

**Table S1** Primers used for multipoint mutagenesis introduction in the study

| **Primers** | **^a^5’🠢3’** |
| --- | --- |
| Y21H−F | actctgccgccgcc*CAC*tgcggcaag |
| Y21H−R | cttgccgca*GTG*ggcggcggcagagt |
| Y21F−F | actctgccgccgcc*TTC*tgcggcaag |
| Y21F−R | cttgccgca*GAA*ggcggcggcagagt |
| F113W−F | acgacggc*TGG*acctcctcct |
| F113W−R | aggaggaggt*CCA*gccgtcgt |
| F113Y−F | acgacggc*TAC*acctcctcct |
| F113Y−R | aggaggaggt*GTA*gccgtcgt |
| F113I−F | acgacggc*ATC*acctcctcct |
| F113I−R | aggaggaggt*GAT*gccgtcgt |
| I202P−F | acccacaccaacgac*CCC*gtcccccgactgcccccccgagagtttggatactcccactcc |
| I202P+F211L−F | acccacaccaacgac*CCC*gtcccccgactgcccccccgagag*CTC*ggatactcccactcc |
| I202P+L206V−F | acccacaccaacgac*CCC*gtcccccga*GTC*cccccccgagagtttggatactcccactcc |
| I202P+L206I−F | acccacaccaacgac*CCC*gtcccccga*ATC*cccccccgagagtttggatactcccactcc |
| I202P+L206V+F211Y−F | acccacaccaacgac*CCC*gtcccccga*GTC*cccccccgagag*TAC*ggatactcccactcc |
| I202V−F | acccacaccaacgac*GTC*gtcccccgactgcccccccgagagtttggatactcccactcc |
| I202L−F | acccacaccaacgac*CTG*gtcccccgactgcccccccgagagtttggatactcccactcc |
| I255A−R | gaattaattcgcggccgcttacaggcaggtgcc*gat*caggccgaagtacca*cag*gtgggcggg*GGC*gtcggggatgttg |
| I255A+L259F−R | gaattaattcgcggccgcttacaggcaggtgcc*gat*caggccgaagtacca*GAA*gtgggcggg*GGC*gtcggggatgttg |
| I255A+L259S−R | gaattaattcgcggccgcttacaggcaggtgcc*gat*caggccgaagtacca*GTA*gtgggcggg*GGC*gtcggggatgttg |
| I255A+L259S−R | gaattaattcgcggccgcttacaggcaggtgcc*gat*caggccgaagtacca*GGA*gtgggcggg*GGC*gtcggggatgttg |
| I255A+L259I−R | gaattaattcgcggccgcttacaggcaggtgcc*gat*caggccgaagtacca*GAT*gtgggcggg*GGC*gtcggggatgttg |
| I255L−R | gaattaattcgcggccgcttacaggcaggtgcc*gat*caggccgaagtacca*cag*gtgggcggg*CAG*gtcggggatgttg |
| I255L+L259F−R | gaattaattcgcggccgcttacaggcaggtgcc*gat*caggccgaagtacca*GAA*gtgggcggg*CAG*gtcggggatgttg |
| I255L+L259Y−R | gaattaattcgcggccgcttacaggcaggtgcc*gat*caggccgaagtacca*GTA*gtgggcggg*CAG*gtcggggatgttg |
| I255L+L259Y+I265L−R | gaattaattcgcggccgcttacaggcaggtgcc*CAG*caggccgaagtacca*GTA*gtgggcggg*CAG*gtcggggatgttg |

^a^ Mutants are underlined in italic capitals

**Table S2** The average values of SASA, Rg, RMSD, and number of hydrogen bonds

| **Wild-type/mutants** | **SASA (nm^2^)** | **Rg (nm)** | **RMSD (nm)** | **hbnum** |
| --- | --- | --- | --- | --- |
| **wt−TLL** | 120.13 ± 0.83 | 1.749 ± 0.005 | 0.239 ± 0.002 | 208 ± 1 |
| **M11** | 116.02 ± 0.57 | 1.737 ± 0.000 | 0.143 ± 0.001 | 214 ± 1 |
| **M21** | 116.76 ± 0.68 | 1.743 ± 0.008 | 0.158 ± 0.007 | 213 ± 1 |

**Table S3** Average contents of secondary structures in wt−TLL and the mutants M11 and M21 determined via MD simulations (100 ns) at 353 K

| **Wild-type**  **/mutants** | **Average values of the content of secondary structures (%)** | | | |
| --- | --- | --- | --- | --- |
|  | **Coil** | **Strand** | **Turn** | **Helix** |
| **wt−TLL** | 21.26 ± 0.91 | 33.14 ± 0.93 | 16.32 ± 0.26 | 29.29 ± 0.24 |
| **M11** | 20.33 ± 0.33 | 33.61 ± 0.36 | 15.37 ± 0.81 | 30.68 ± 0.12 |
| **M21** | 20.87 ± 1.31 | 33.99 ± 1.27 | 15.28 ± 1.70 | 29.86 ± 1.74 |

**Table S4** The docking binding energies for selected docking conformations calculated by molecular docking

| **Wild-type**  **/mutants** | **Binding energy (kcal/mol)** | | | |
| --- | --- | --- | --- | --- |
|  | ***p*NPP** | **palmitic acid** | **oleic acid** | **linoleic acid** |
| **wt−TLL** | −4.27 | −4.66 | −4.29 | −14.68 |
| **M11** | −4.54 | −5.23 | −4.9 | −16.67 |
| **M21** | −4.62 | −4.84 | −4.63 | −15.79 |


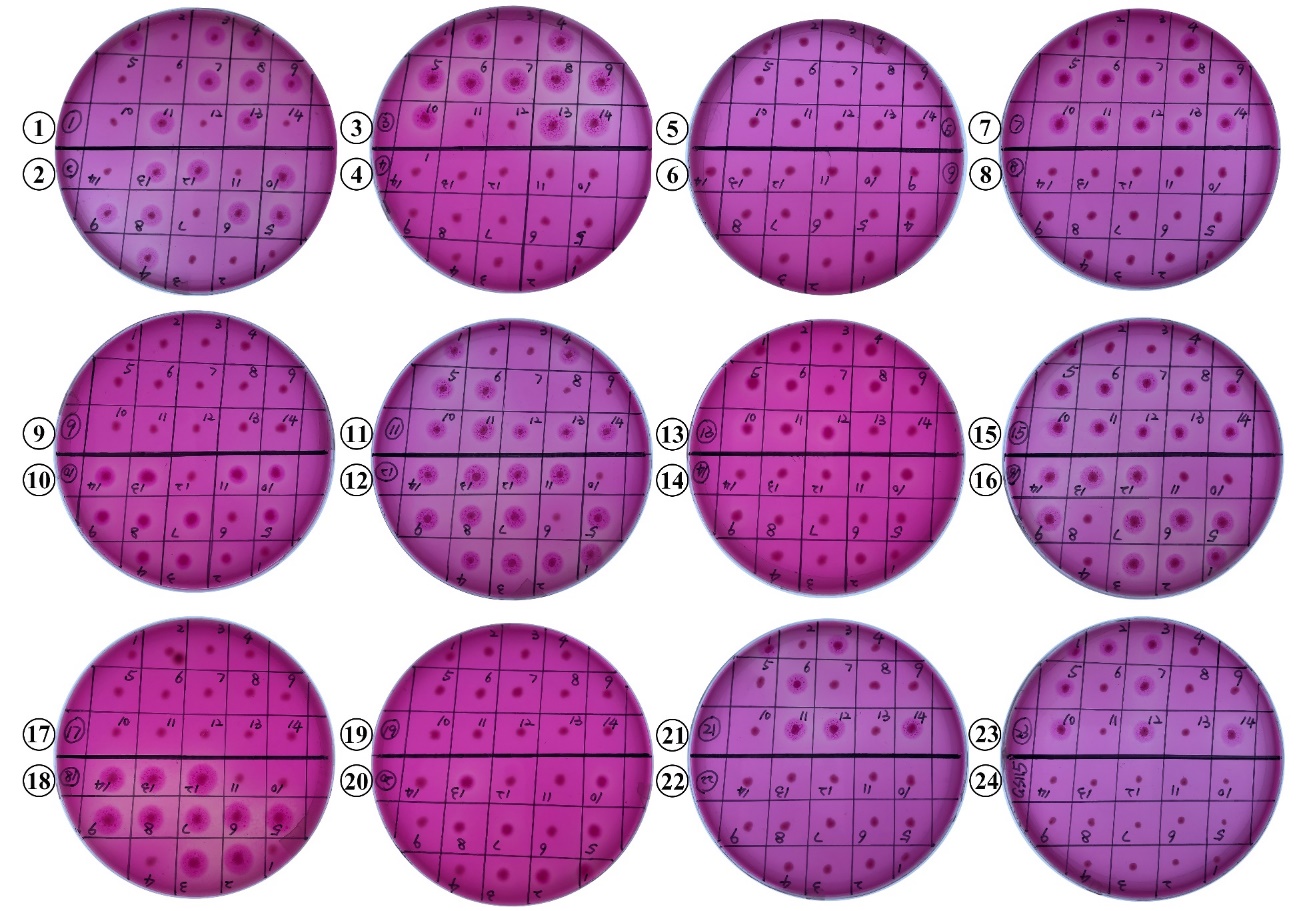


**Fig. S1** Screening of lipolytic activity of recombinant TLL on rhodamine B−olive oil agar plate under visible light. (1) mutant M01, (2) mutant M02, (3) mutant M03, (4) mutant M04, (5) mutant M05, (6) mutant M06, (7) mutant M07, (8) mutant M08, (9) mutant M09, (10) mutant M10, (11) mutant M11, (12) mutant M12, (13) mutant M13, (14) mutant M14, (15) mutant M15, (16) mutant M16, (17) mutant M17, (18) mutant M18, (119) mutant M19, (20) mutant M20, (21) mutant M21, (22) mutant M22, (23) wt−TLL, (24) parent *Pichia pastoris*, GS115.


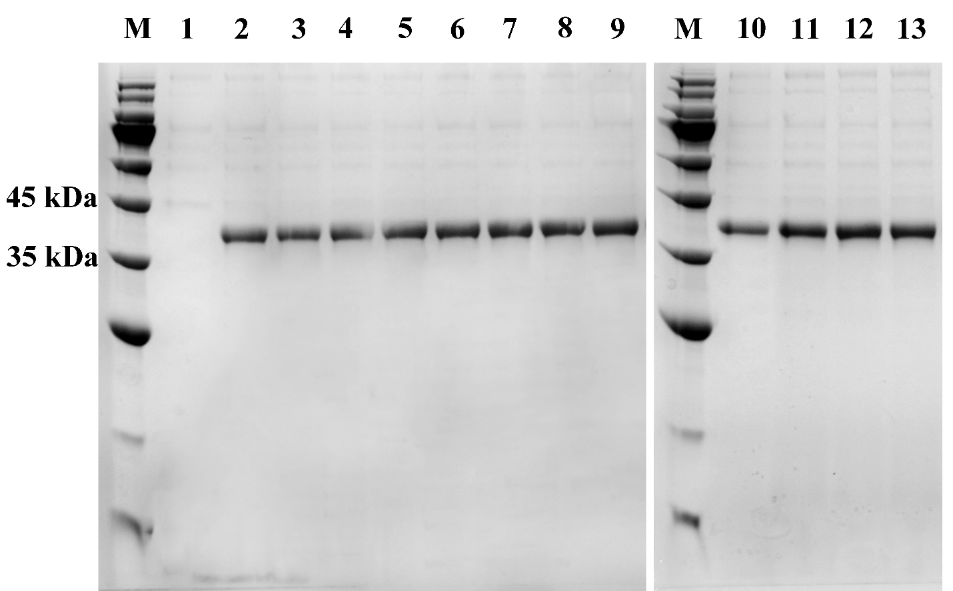


**Fig. S2** Determination of protein molecular weight of wild and mutant TLL on SDS‑PAGE using 15% precast mini polyacrylamide gel. Lane M: standard protein molecular weight (180/140/100/75/60/45/35/25/15/10 kDa), lane 1: parent *P. pastoris* GS115, lane 2: mutant M01, lane 3: mutant M02, lane 4: mutant M03, lane 5: mutant M07, lane 6: mutant M10, lane 7: mutant M11, lane 8: mutant M12, lane 9: mutant M15, lane 10: mutant M16, lane 11: mutant M18, lane 12: mutant M21, lane 13: wt−TLL.


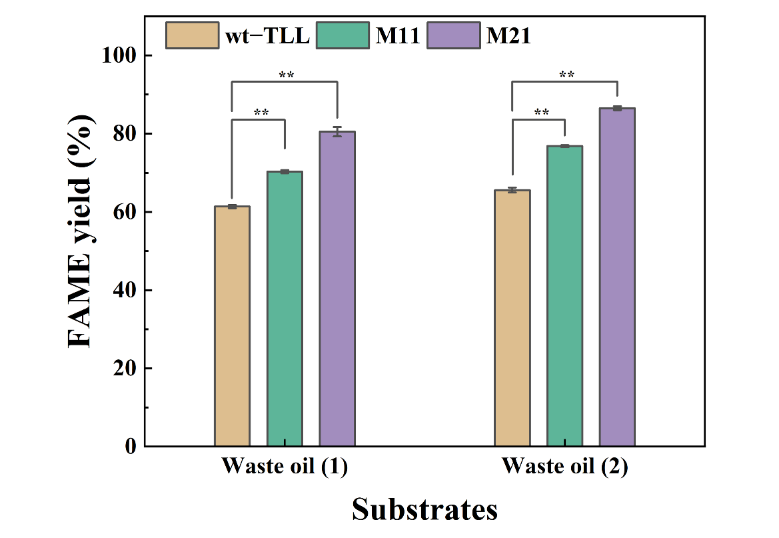


**Fig. S3** Biotransformation of waste oils catalyzed to FAME by wt–TLL and positive mutants (M11 and M21) for 48 h. The error bars in graphs represent standard deviations (n = 3). The asterisks indicate significant differences between mutants and wild-type: *, *p* < 0.05; **, *p* < 0.01.


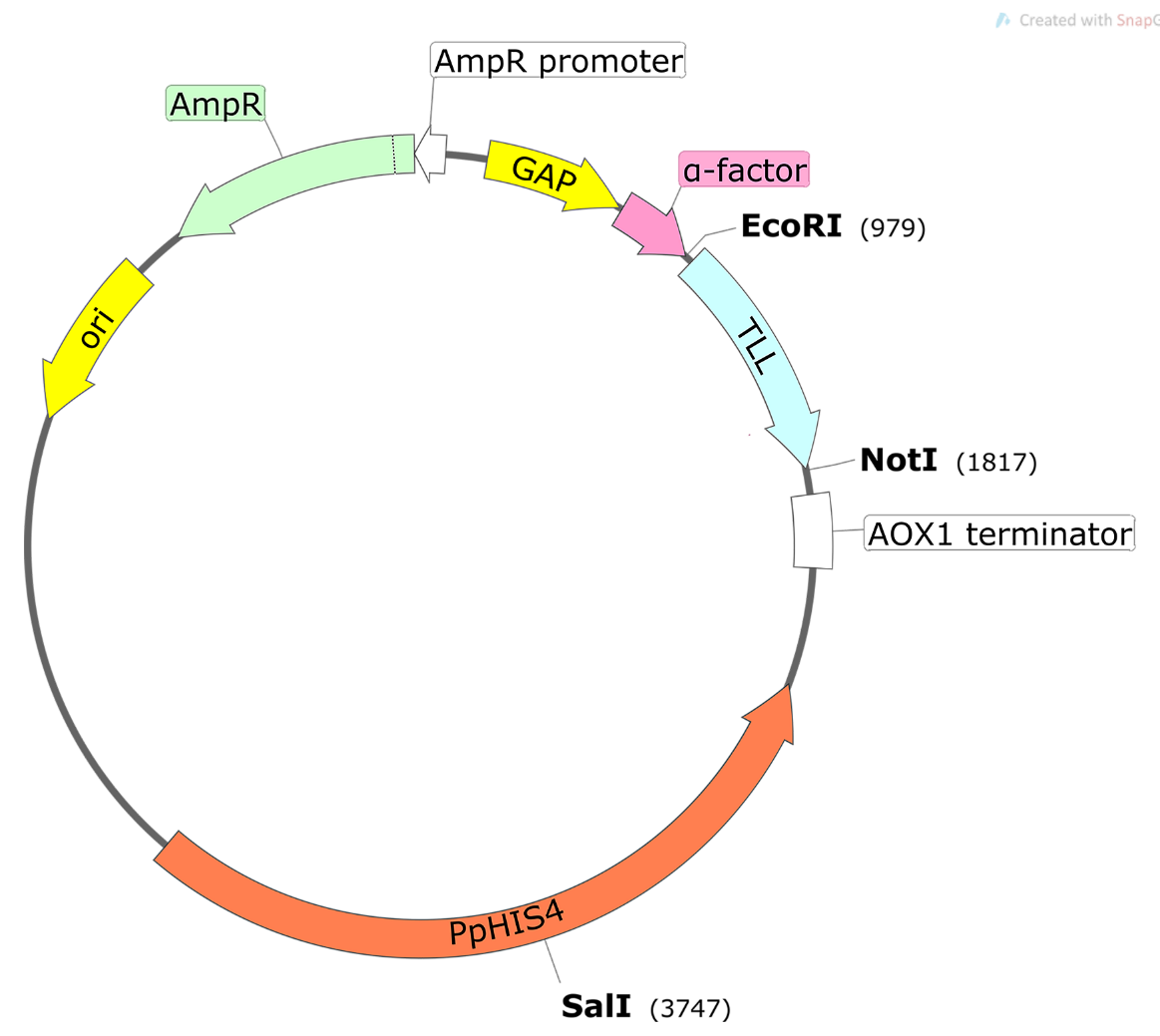


**Fig. S4** The schematic diagram of recombinant plasmid pGAP815α/TLL including TLL gene with 6×His‑tag in N‑terminal, GAP promoter and an evolved signal peptide from yeast *Saccharomyces cerevisiae*.
